# Meeting Report: International Symposium on the Genetics of Aging and Life History II

**DOI:** 10.18632/aging.100762

**Published:** 2015-06-15

**Authors:** Murat Artan, Ara B. Hwang, Seung‐Jae V. Lee, Hong Gil Nam

**Affiliations:** ^1^ Information Technology Convergence Engineering, Pohang University of Science and Technology, Pohang, Gyeongbuk, South Korea; ^2^ Department of Life Sciences, Pohang University of Science and Technology, Pohang, Gyeongbuk, South Korea; ^3^ School of Interdisciplinary Bioscience and Bioengineering, Pohang University of Science and Technology, Pohang, Gyeongbuk, South Korea; ^4^ Center for Plant Aging Research, Institute for Basic Science, and Department of New Biology, DGIST, Daegu, South Korea

**Keywords:** aging, life history, longevity, South Korea, meeting report

## Abstract

The second International Symposium on the Genetics of Aging and Life History was held at the campus of Daegu Gyeongbuk Institute of Science and Technology (DGIST), Daegu, South Korea, from May 14 to 16, 2014. Many leading scientists in the field of aging research from all over the world contributed to the symposium by attending and presenting their recent work and thoughts. The aim of the symposium was to stimulate international collaborations and interactions among scientists who work on the biology of aging. In the symposium, the most recent and exciting work on aging research was presented, covering a wide range of topics, including the genetics of aging, age‐associated diseases, and cellular senescence. The work was conducted in various organisms, including *C. elegans*, mice, plants, and humans. Topics covered in the symposium stimulated discussion of novel directions for future research on aging. The meeting ended with a commitment for the third International Symposium on the Genetics of Aging and Life History, which will be held in 2016.

## INTRODUCTION

Aging is a fundamental problem that the world is currently facing. The population of elderly people is higher than it has ever been before and continues to increase at an even higher rate. Although life expectancy has been dramatically increased in industrialized countries, many elderly people still suffer from serious age-related diseases, and the burden of healthcare costs is increasing steadily because aging is directly related to many illnesses, including cancer, diabetes, and cardiac dysfunction. Therefore, delaying the onset of age-related diseases, improving quality of life, and providing humans with a healthy aging strategy are among the main goals of research on aging. Model organisms have proven to be reliable tools for studying aging and have revealed promising biological foundations for delaying the onset and the progress of age-related human diseases.

**Figure 1:**
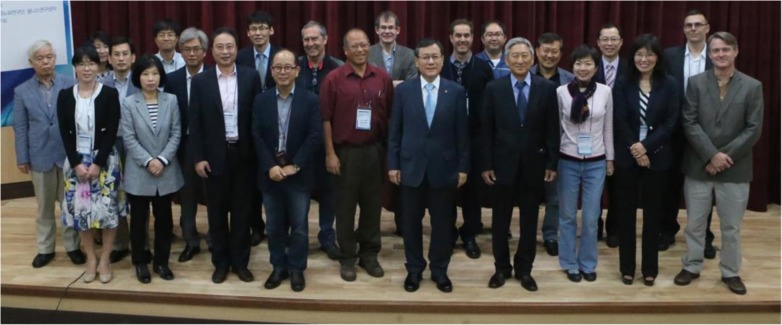
The organizing committee members and the speakers at the second International Symposium on the Genetics of Aging and Life History, which was held from May 14 to 16, 2014 at the campus of DGIST, Daegu, South Korea.

To address and discuss emerging issues on various aspects of the biology of aging, the International Symposium on the Genetics of Aging and Life History II was held at the campus of the Daegu Gyeongbuk Institute of Science and Technology (DGIST), South Korea, from May 14 to 16, 2014. Many leading scientists from all over the world attended the symposium to present their work and to share their ideas (Fig. 1). The meeting kicked off with a grand welcome from the president of DGIST, Dr. Sung-Chul Shin, and from the advisory committee chair, Dr. Bub Wan Kim. The meeting was continued by 18 scientists presenting their work focusing on various topics in aging, such as the genetics of aging, age-associated diseases, and cellular senescence. The organisms used in their research ranged widely, from *C. elegans* to plants and humans. Junior scientists and graduate students also had an opportunity to present their work at a poster presentation session and to interact with the invited speakers.

### Mitochondrial signaling

Mitochondria are essential organelles that regulate several core biological processes and are closely linked to aging. Interestingly, mild inhibition of mitochondrial respiration promotes longevity in the nematode *C. elegans*. Recent findings suggest that ROS generation by mitochondria is a part of the mechanism that helps cells adapt to the aging process [1, 2]. At this meeting, Dr. Siegfried Hekimi (McGill University, Canada) presented his recent work proposing one of the mechanisms by which ROS affect the aging process [3]. Surprisingly, this mechanism involves the intrinsic apoptotic pathway, which helps sense, kill, and remove cells when they are damaged beyond repair and need to be replaced. His group found that when the apoptosis pathway is stimulated by ROS in *C. elegans*, the longevity of the organism as a whole is increased. Notably, removal of damaged cells is not an option in post-mitotic nematodes, where each cell carries out an irreplaceable function. These findings could guide the development of therapeutic options to prevent or fight age-dependent neurodegenerative diseases, as simple removal of damaged cells is also not an option in the brain.

Dr. Ara B. Hwang in the laboratory of Dr. Seung-Jae V. Lee (Pohang University of Science and Technology, South Korea) presented their work elucidating a feedback regulatory mechanism and the functional significance of ROS-induced longevity. In a previous report [4], they demonstrated that increased hypoxia-inducible factor 1 (HIF-1) activity caused by moderately elevated ROS levels mediates the longevity of a mitochondrial respiratory mutant of *C. elegans*. At this meeting, they showed that activation of adenosine monophosphate (AMP)-activated protein kinase (AMPK) as well as HIF-1 mediates the longevity response to ROS [5]. Furthermore, AMPK and HIF-1 act as positive and negative feedback regulators of ROS, respectively. Their new study imply that this feedback regulation of ROS enhance immunity and thereby contribute to longevity.

Another signaling pathway that is required for the long lifespan of mitochondrial respiratory mutants is the mitochondrial unfolded protein response (UPR^mito^) [6]. It is already known that the mitochondria, cytosol, and endoplasmic reticulum (ER) are each equipped with well-compartmentalized specific stress-response systems to deal with unfolded or misfolded proteins [7]. Dr. Hyun-Eui Kim in the laboratory of Dr. Andrew Dillin (Howard Hughes Medical Institute at the University of California at Berkeley, USA) presented their work on compartmental communication in *C. elegans* stress responses. They showed that activating UPR^mito^ greatly improves cytosolic protein homeostasis via a novel signaling pathway that affects metabolic pathways. The activation of this signaling pathway protects the organism from proteotoxicity induced by aggregate-prone disease-associated proteins. Their study proposes a novel mechanism that links mito-chondrial protein homeostasis to other cellular compartments.

### Sensory signaling and aging

How environmental stimuli affect the aging process is another question that many scientists have asked. Studies using *C. elegans* and *Drosophila* have clearly showed that environmental cues such as smell and temperature affect lifespan [8]. In *C. elegans*, mutations in a subset of sensory neurons increase the lifespan, and this requires the activation of the *daf-16*/FOXO longevity transcription factor [9]. Dr. Seung-Jae V. Lee presented his work on the role of the sensory system in lifespan regulation in *C. elegans*. He showed that several insulin-like peptides act as neuroendocrine factors that transmit lifespan-regulatory signals from sensory neurons to distal tissues. These insulin-like peptides regulate the transcriptional activity and nuclear localization of the longevity factor *daf-16*/FOXO. His study provides an endocrine link between the sensory system and FOXO-mediated lifespan regulation to better illustrate how sensory inputs affect longevity in *C. elegans*.

Dr. Andrew Dillin addressed whether the perception of pain elicits long-term consequences for the organism. Clinical studies suggest a correlative decrease in lifespan and overall decrease in the quality of health in patients that experience chronic levels of pain [10]. Dr. Dillin provided evidence that mutations in TRPV1 (transient receptor potential cation channel subfamily V member 1; receptors for capsaicin and noxious stimuli) extend lifespan in mice and worms through a neuroendocrine pathway [11]. TRPV1-expressing neurons regulate lifespan through cAMP response element binding protein (CREB), which modulates the secretion of CGRP (calcitonin gene-related peptide) neuropeptides. CGRP antagonizes insulin secretion from pancreatic β cells. Moreover, pharmacological inhibition of CGRP increases metabolic health at the later stages of life. From this work, Dr. Dillin reported the discovery of a novel neuroendocrine circuit that represses longevity by adjusting metabolic activity through CRTC1/CREB circuit.

### Nutrients and aging

Although nutrients are essential for the regulation of many physiological processes and are required for organismal survival, excessive caloric intake can lead to diabetes, cardiovascular disease, and certain forms of cancer [12]. Carbohydrates are not an exception to this, and it is well established that increased glucose accelerates aging in yeast and *C. elegans* [12]. A high-glucose diet shortens the lifespan of worms by down-regulating the activity of AMPK, *daf-16*/FOXO, and glyoxalase [13–15]. In the second part of his talk, Dr. Seung-Jae V. Lee described his group's recent study regarding how lipid synthesis counteracts the life-shortening effects of a glucose-rich diet in *C. elegans*. They showed that promoting the synthesis of unsaturated fatty acids protects worms from the deleterious effects of dietary glucose. Their work may provide insights into the mechanisms by which key nutritional factors such as carbohydrates influence aging.

Dae Won Moon (DGIST, South Korea) presented his work on multimodal coherent anti-stokes Raman scattering (CARS) microscopy and secondary ion mass spectrometry (SIMS), label-free imaging techniques applied to *C. elegans* and the olfactory bulbs of mice. By using CARS, images of lipids in *C. elegans* were visualized with 300 nm resolution. Dr. Moon also presented his group's data comparing total amounts of lipids in wild-type and mutant *C. elegans*. He further showed that SIMS technology can be used to visualize specific lipid molecules, including fatty acids, glycerolipids, glycerophospholipids, choresterol, and neurotransmitters, including dopamine, glutamate, gamma-aminobutyric acid (GABA), acetylcholine, and pheromones. Thus, his studies may provide a new platform technology for visualizing aging processes and the relationship between *in situ* nutrients and their derivatives.

### MicroRNAs and aging

MicroRNAs (miRNAs) regulate biological processes, including aging, by influencing gene expression. Dr. Frank Slack (Harvard University, USA) presented results from his laboratory's studies that determine the role of microRNAs in aging in *C. elegans* [16]. From a small-scale RNA sequencing analysis, they identified multiple miRNAs that are expressed differentially between young and old animals. Knockout experiments revealed that some of these miRNAs are required for a normal lifespan in *C. elegans*. Some are also required for the lifespan extension caused by dietary restriction. A systems analysis placed these miRNAs in known pathways of aging.

Dr. Keetae Kim (DGIST, South Korea) presented his work on the role of miRNAs in mouse hippocampal aging. He identified various miRNAs that are upregulated during hippocampal aging using small RNA sequencing. He reported that the Eph/ephrin signaling pathway is significantly down-regulated during normal adult aging in the hippocampus. One of the up-regulated miRNAs in the aged hippocampus targets EphB2 and causes a reduction in the EphB2 protein level in hippocampal neurons. This in turn leads to a significantly decreased dendritic spine density in cultured hippocampal neurons. These results suggest that miRNAs play an important role in age-dependent regulation of the Eph/ephrin signaling pathway.

### Human aging

Based on research using model organisms, studies that have extended the scope of our understanding of human aging were also presented at the meeting. Dr. Stuart Kim (Stanford University, USA) discussed the process of aging in the human kidney and *C. elegans*. He asked whether there is an underlying intrinsic clock for aging. He presented results showing that the expression levels of several transcription factors change with age. This leads to a cascade of changes in expression of genes in several organs, which results in dysfunction and physiologic breakdown. By manipulating the age-related transcription factors, his group was able to show that these transcription factors were able to molecularly reprogram gene expression patterns from the old to the young state, resulting in a beneficial physiology associated with youthfulness.

Centenarians, those who live more than a century, have been human models for exceptional longevity. Genes involved in the evolutionarily conserved pathways that have major impacts on lifespan in animal models have been prime candidates in efforts to identify naturally occurring genetic variations associated with human longevity [17]. These include insulin/insulin-like growth factor-1 (IGF-1) signaling. Dr. Yousin Suh (Albert Einstein College of Medicine, USA) previously discovered two centenarian-enriched missense mutations in the IGF-1 receptor gene (*IGF1R*), A37T (M1) and A407H (M2), that are associated with decreased insulin/insulin-like growth factor signaling (IIS) in lymphocytes from carriers as compared to non-carriers [18]. To elucidate the molecular mechanisms by which these variants promote longevity in humans, they have generated a knock-in mouse model expressing the longevity-associated human *IGF1R* M2 variant. Dr. Suh's laboratory is currently characterizing the changes in IIS induced by a high-fat diet in the *IGF1R* knock-in animals. Her studies will define the functional consequences of human longevity-associated *IGF1R* mutations. Her studies also provide invaluable insights into the mechanistic relationship between reduced IIS and longevity in humans, as well as revealing *in vivo* targets of IIS for intervention strategies.

Aging is associated with many complex diseases. Reliable prediction of the aging process is important for assessing the risks of aging-associated diseases. However, despite intense research, so far there is no simple and reliable aging marker. Dr. Jing-Dong Jackie Han (Shanghai Institutes of Biological Sciences, Chinese Academy of Science, China) tackled this problem by examining whether non-invasive imaging of human three-dimensional (3D) facial features can be used as reliable aging markers [19]. She collected more than 300 3D-facial images and blood profiles across individuals aged 17−77 years. By analyzing their morphological profiles, Dr. Han and her colleagues performed the first comprehensive mapping of the aging human facial phenome, and found that quantitative facial features such as the eye slope and mouth-nose distance are highly associated with age. They constructed a robust age predictor and identified fast and slow aging outliers; these predictions are significantly supported by health indicators in blood [19]. Thus, facial features are reliable aging biomarkers that reflect the general health status of an individual rather than the chronological age.

Diabetes is a well-known aging-associated disease. Extracellular matrix (ECM) components play important roles in controlling the survival and function of β cells and islets. Dr. Won Bae Jeon (DGIST, South Korea) used an RGD (arginine-glycine-aspartate)-modified elastin-like polypeptide (REP) as a bioactive matrix to improve age-induced β cell malfunction. Compared with young mice, old mice display oral glucose tolerance, a decrease in glucose-stimulated insulin secretion, and reduced expression of ECM proteins (fibronectin, laminin, and collagen I and IV) in the islets. They showed that treatment with REP and ECM proteins improves the viability of islets and activates both extracellular signal-regulated kinase (ERK) and protein kinase B (Akt). This treatment also up-regulates insulin, BETA2, and the transcription factor pancreatic and duodenal homeobox 1 (PDX-1), and elevates glucose-stimulated insulin secretion. His study demonstrated that modulating ECM components can be an effective strategy for improving the dysfunction of aged islets.

### Models of genetic diseases that affect aging

Abnormal splicing of the *LMNA* gene, which encodes nuclear lamina protein lamin A, or prelamin A, results in the expression of a truncated lamin A protein called progerin, which leads to Hutchinson–Gilford progeria syndrome [20, 21]. Progerin has a compromised regulatory function in chromatin remodeling, leading to defective DNA repair and accelerated aging. In his talk, Dr. Zhongjun Zhou (University of Hong Kong, Hong Kong) reported that lamin A activates the histone-modifying enzyme sirtuin 1, thereby modulating the chromatin conformation [22]. Sirtuin 1 exhibits reduced association with the nuclear matrix (NM) and decreased deacetylase activity, which results in altered histone modifications in the presence of progerin or prelamin A. This leads to rapid depletion of adult stem cells (ASCs) in progeroid mice. Resveratrol, a sirtuin 1 activator, enhances the binding of A-type lamins to sirtuin 1 to increase its deacetylase activity. Dr. Zhou further showed that in progeroid mice, resveratrol treatment rescues ASC decline and significantly extends lifespan. These data suggest that lamin A is an endogenous activator of sirtuin 1. In addition, his work suggests a potential stem cell-based and sirtuin 1 pathway-dependent therapeutic strategy for progeria.

Defects in mitochondrial respiration caused by mutations in genes encoded by the nuclear or mitochondrial DNA are closely associated with human diseases [23]. Severe mitochondrial disorders also lead to a very short life expectancy in several organisms. Dr. Matt Kaeberlein (University of Washington, USA) described his group's efforts to reveal the relationship between mitochondrial dysfunction and normative aging. He discussed their recently published work showing that the mechanistic target of rapamycin (mTOR) inhibitor rapamycin is effective at attenuating disease progression in the *Ndufs4^−/−^* mouse model of the childhood mitochondrial disorder Leigh Syndrome [24]. Their unpublished data suggested new pharmacological interventions, which may also be effective in this disease model. He then presented data from ongoing mid-life intervention trials to determine whether a short-term regimen of high-dose rapamycin can significantly improve lifespan and healthspan in aged mice.

### Cellular senescence

Cellular senescence is a stress-response phenomenon in which cells lose the ability to proliferate. Cellular senescence is induced by telomere erosion, activation of oncogenes or tumor suppressor genes, or exposure to a sub-lethal dose of DNA damaging agents or oxidative stresses. These stimuli activate the p53/pRb pathways, the DNA-damage response, and autophagy [25]. This leads to extensive chromatin remodeling and senescence-associated secretion of various factors. Cellular senescence plays an important role in tumor suppression, wound healing, and organismal aging, as well. Dr. Peter Adams (Beatson Institute for Cancer Research and the University of Glasgow, United Kingdom) presented his work on two topics regarding chromatin dynamics and healthy aging. First, he presented data on genome-wide sequencing to investigate the patterns of DNA methylation in senescent cells [26]. His group found that senescent cells display widespread hypomethylation and focal hypermethylation similar to that seen in cancer cells. These features are also largely retained when cells bypass senescence. He speculated that senescence-associated epigenetic changes may contribute to the onset of age-associated diseases by altering chromatin structure. Dr. Adams also presented data showing that senescent cells actively exploit dynamic chromatin structure to enact an efficient senescence-associated tumor suppression [27]. However, accumulation of senescent cells in tissues during aging may trigger age-related tissue dysfunction and diseases, including cancer. Their work provided insights into the role of chromatin control in regulating both the detrimental and beneficial effects of cellular senescence on aging and the onset of age-related diseases.

Inflammation is an underlying basis for the molecular alterations that link aging and age-related pathological processes. Mitotic dysregulation is implicated in cellular aging. Dr. Jae-Ryong Kim (Yeungnam University, South Korea) presented his work showing that insulin-like growth factor binding proteins (IGFBP-3 and IGFBP-5) and interferon gamma (IFN-γ) accelerate cellular senescence through a p53-dependent DNA damage signaling pathway [28-30]. In addition, inflammatory mediators, such as phospholipase A2 and prostaglandin, induce senescence phenotypes in human cells [31, 32]. Conversely, the mitotic proteins Aurora kinase B (AURKB) and polo-like kinase 1 (PLK1) inhibit cellular senescence via the p53 pathway [33, 34]. These results suggest that a variety of secretory molecules, inflammatory mediators, and mitotic proteins play important roles in cellular senescence through the p53 pathway and contribute to the pathogenesis of age-related diseases.

### Plant aging and senescence

In plants, “senescence” is the term favored over “aging”. Plant senescence is one of the most prevalent developmental events in nature, as seen in the grand scenery of autumn leaves or in the rice or wheat fields. It is an evolutionarily acquired process critical for plants’ fitness. The process of leaf senescence provides energy and materials to the growing parts of the plant or developing seeds by disassembling and transporting nutrients acquired during the growth season. Plant senescence occurring at the organismal level may negatively affect the fitness at the individual level but the nutrient relocation mechanisms during the death of whole plants ensures energy and materials necessary for developing progeny, thus increasing the fitness at the population level.

In his talk, Dr. Hong Gil Nam (DGIST) presented the molecular mechanisms of developmental transitions along the lifespan during Arabidopsis aging. His group has been unraveling the regulatory mechanisms underlying how plants decide to senesce at the organ and organismal levels at a given age, mainly by identifying and studying mutants with altered organ senescence. From these studies, they previously identified the trifurcate feed-forward pathway for age-dependent cell death, which revealed a unique aspect of aging and senescence control mechanisms in plants [36]. They further investigated changes in transcriptome along the lifespan through total and small RNA sequencing approaches and interestingly found that senescence stage involves a more coherent regulation of transcriptome. Dr. Nam's group is also trying to reveal temporal transitions and interactions of the regulatory networks composed of the plant NAC (NAM, ATAF, and CUC) transcription factors. Dr. Nam's group launched the Center for Plant Aging Research, Institute for Basic Science and is now pursuing novel aspects of plant aging, such as integration of lifespan phenome with molecular - omics, mechanism of heterogeneous cell death during senescence, discovery towards the aging clock, interaction between circadian clock and aging, evolution of aging process using ecotypes, mechanisms of systemic whole plant senescence, and role of nutrient relocation in plants.

### Conclusions

The first International Symposium on the Genetics of Aging and Life History was held on the Pohang University of Science and Technology (POSTECH) Campus, Pohang, South Korea, in 2012. The second meeting at DGIST was also very successful, and was also very beneficial for both Korean and international attendees. The success of the first two meetings clinched the enthusiasm of the organizing committee for the next meeting, which will be held in 2016.

The scientists at the meeting presented their work, which aims to identify solutions for aging and age-associated diseases. Pharmacological strategies and bioinformatics approaches to understand aging, cellular senescence, sensory and mitochondrial signaling, and the role of microRNAs in the regulation of lifespan were among the wide range of topics covered at the meeting. Interventions that slow aging and delay the onset of age-associated diseases were discussed thoroughly.

There is no doubt that the importance of research on aging has been emphasized in the last few decades. The increasing interest in and demand for aging research is also felt in East Asian countries, including South Korea. Many notable meetings on aging research have been successfully held in Asia, including the Symposium on the Genetics of Aging and Life History (South Korea), the Trinations Aging Symposium (China) [37], a conference on the Molecular Basis of Aging and Disease, Cold Spring Harbor Laboratory Asia (China), and others. Leading scientists in the field are invited from throughout the world, and the number of participants has been increasing at each meeting. We believe that these meetings, including the Symposium on the Genetics of Aging and Life History, substantially bridge different areas of aging research and bring opportunities for collaborations. As Dr. Kaeberlein and Dr. Sang Chul Park (Director of the Samsung Well Aging Research Center, South Korea) commented during their overview talk and advisory comments, respectively, the emergence of an aging society is an issue of the utmost importance in many developed countries, and we hope that this meeting will help achieve the eventual goal of healthy aging in humans.
